# The impact of social participation on Subjective Wellbeing in the older adult: the mediating role of anxiety and the moderating role of education

**DOI:** 10.3389/fpubh.2024.1362268

**Published:** 2024-05-16

**Authors:** Zaihua Qing, Caihong Wu, Tao Gao

**Affiliations:** ^1^Hunan University of Finance and Economics, Changsha, China; ^2^Guangdong Industry Polytechnic, Guangzhou, China

**Keywords:** social participation, subjective Wellbeing, education, Chinese older adult, mediating role, moderating role

## Abstract

**Introduction:**

The study aims to examine the mediating role of anxiety in the relationship between social participation and Subjective Wellbeing among Chinese older adults. Additionally, it investigates the moderating ed of education in this relationship.

**Methods:**

The data came from the Chinese Longitudinal Healthy Longevity Survey (CLHLS) published by peking University, with a sample size of 10,626 individuals aged 60 years and above. SPSS 21.0 was used for the statistical analysis of the data, and Mplus 8.0 was used for the statistical processing of the mediating and moderating effects analysis.

**Results:**

(1) The social participation significantly and positively predicated Subjective Wellbeing; (2) Anxiety partially mediated the eect between social participation and Subjective Wellbeing. The mediating eect value was 0.103; (3) Education plays a moderating role in the impact of social participation on subjective Wellbeing.

**Discussion:**

In summary, social participation can reduce the anxiety and enhance their Subjective Wellbeing. Meanwhile, the eet of social participation on Subjective Wellbeing was the greatest for the older adult with high education. The findings suggest that community-led activities can be initiated to improve social participation in the older adult. Furthermore, educational courses could be to support the healthy aging of older adults in China.

## 1 Introduction

The aging of the population is a worldwide social phenomenon. With the proportion of aging population continually on the rise, aging has become a fundamental national concern in China. By 2050, it is estimated that China's older adult population aged 65 and above will account for 25.6% of the total population, and society will reach a stage of deep aging (1). As the older adult population continues to expand, their need for health services has become increasingly urgent. The older adult's living conditions, quality of life, and other factors have received widespread attention from all sectors of society. The older adult population is generally more susceptible to diseases and requires more care. Therefore, as population aging accelerates, the government has proposed the strategic goal of active aging to improve the existing policy protection with the perspective of healthy aging (2, 3). Healthy aging is mainly assessed by the quality of life in the older adult, and the Subjective Wellbeing of the older adult is an important indicator to evaluate the healthy aging process. Helping the older adult improve their quality of life has become a salient social phenomenon (4, 5).

Subjective Wellbeing, also known as psychological Wellbeing, is primarily an individual's overall assessment of their quality of life based on their self-set criteria (6). It is reflected in the individual's life satisfaction, the acquisition of positive emotions, and the disappearance of negative emotions, and it is characterized by subjectivity, wholeness, and relative stability (7). According to existing studies, higher Subjective Wellbeing is associated with reduced risks of cardiovascular disease, cognitive decline, physical frailty, and mortality in the older adult (8, 9). Therefore, studying the Subjective Wellbeing of the older adult and improving their quality of life bear great social and practical significance.

Social participation is a contributing factor to healthy aging. It refers to a pattern of behavior in which participants realize their own values in the process of social interaction in the form of social work or social activities (10). As per the theory of the older adult subcultural group, the older adult fulfill their inner psychological needs in the process of social participation and promote communication among them. The older adult feel relaxed in the subcultural group, can obtain happiness, form a positive mindset, and obtain a sense of satisfaction in life (11). Social participation can reduce the older adult's frailty, improve their memory, and reduce depression. By participation, the older adult can create social value and enhance their own sense of Wellbeing (12–14). Therefore, this study proposes hypothesis 1: Social participation would positively predict the older adult's Subjective Wellbeing, Social participation may increase the older adult's Subjective Wellbeing.

Anxiety significantly impacts the Wellbeing of the older adult. Research has shown that there is a significant negative correlation between anxiety and happiness. The greater the severity of anxiety experienced by the older adult, the lower the level of happiness they perceive (15). As they age, older adult individuals experience degenerative physiological changes that affect their daily living and self-care abilities. Moreover, coupled with the onset of their diseases, retirement, social disconnection, and fear of death, all make the older adult feel lonely, helpless, and anxious (16, 17). These negative emotions can affect the older adult individuals' feelings and evaluation of their lives, thus affecting their perceived Wellbeing. The Activity Theory posits that the older adult should actively participate in society to replace the social roles they have lost due to retirement, and so on. This can alleviate negative emotions caused by the interruption of social roles, thereby reducing their societal disconnect (18). There have been studies that found that social participation was able to reduce anxiety in the older adult (19). Therefore, the second hypothesis was raised:

Hypothesis 2: Anxiety mediates the relationship between social participation and Subjective Wellbeing.

Hypothesis 2a: Social participation would negatively predict the anxiety of the older adult.

Hypothesis 2b: Anxiety would negatively predict subjective Wellbeing of the older adult.

The educational level of the older adult affects their Subjective Wellbeing, and numerous scholars have argued that education has a beneficially enhances the Wellbeing of the older adult (20, 21). The older adult who is educated is more willing to participate in social activities after retirement, such as physical exercise, leisure activities, etc. The cognitive enrichment hypothesis suggests that education is an important factor in the construction of socio-spiritual resources (22). Compared to individuals with low education level, the older adult with high education are more capable of building social support networks. They increase the frequency of socializing with others in their lives, gain more human capital and development opportunities in society, and display a great willingness to participate in social activities (23). While participating in social activities, they can release stress and negative emotions, maintain a good psychological condition, and increase Subjective Wellbeing (24, 25). Additionally, some studies have revealed that education moderates the relationship between social status and the older adult's Wellbeing (26). To some extent, social participation also reflects the older adult's level of social status. Here we put forward the third hypothesis:

Hypothesis 3: education plays a moderating role in the relationship between social participation and the older adult's Subjective Wellbeing. Education may function as a positive moderator which amplifies the enhancing effect of social participation on subjective Wellbeing.

In conclusion, this study aimed to investigate the mechanisms of social participation on the older adult Subjective Wellbeing, including the mediating role of anxiety and the moderating role of education, in order to provide a scientific basis for enhancing the healthy aging of the older adult.

## 2 Methods

### 2.1 Study population and data source

The data used in this paper were all obtained from the 2018 data from the Chinese Longitudinal Healthy Longevity Survey (CLHLS) published by Peking University. The CLHLS survey's covered 23 provinces and regions, and the total population of the regions involved accounted for approximately 85% of the national. The questionnaire contained information on various aspects, including demographic and sociological characteristics, family background, economic status, health status, and living conditions, which provided better data support for this study. In our pursuit of robust and reliable data, any instances featuring randomly occurring missing values were eliminated from the study. This resulted in a final sample size of 10,626, exclusively composed of individuals aged 60 and above, aligning with our focus on geriatric psychology. The age range within the sample is quite substantial, extending from 60 to 117 years, with an average age of 83.39 years.

### 2.2 Measurements

#### 2.2.1 Social participation

Under the social participation condition, the CLHLS questions on social participation included: outdoor activities (including tai chi, square dancing, hanging out, living with friends, and other outdoor activities), playing cards or mahjong, watching TV or listening to radio, reading books and newspapers, and participating in organized social activities. For each activity, there were five options: almost every day, at least once a week, at least once a month, sometimes, and not at all. The questions were reverse coded and scored on a 5-point scale, with 1 = not participating and 5 = almost every day. The scores for each item were summed to give a total score, with higher scores indicating higher levels of social participation. In the present study, the Cronbach α coefficient for the scale was 0.73.

#### 2.2.2 Generalized anxiety disorde-7

The GAD-7 (27) is a self-rating scale that assesses the frequency of symptoms in the last 2 weeks and can be used to screen for generalized anxiety and its severity. The GAD-7 consists of 7 items, each of which is scored on a 4-point scale. A score of 0 was assigned to “never,” and a score of 3 was assigned to “almost every day.” The total score (0–21) is calculated by adding the scores of each item, with higher scores indicating more severe anxiety. A total score of < 5 was considered as no anxiety symptoms (negative), and ≥5 was considered as having anxiety symptoms (positive), and the Cronbach α coefficient of this scale in this study was 0.92.

#### 2.2.3 Subjective Wellbeing

Some scholars suggest that Subjective Wellbeing should include an emotional component (mainly the presence of positive emotions and the absence of negative emotions) and an evaluative component (life satisfaction) (6, 28, 29). This paper addresses six questions in the CLHLS regarding the evaluation of the older adult's life and the older adult's emotional state. The questions were, “In general, how do you think you are living your life?” “Are you hopeful about your future life?” “Are you as happy now as you were when you were younger?” “Do you often feel nervous or scared?” “Do you often feel lonely?” “Do you feel less and less useful as you get older?” Each of the six questions had five options in the order of 1 to 5, with 1 being very happy or always and 5 being very unhappy or never. The above six questions were reverse coded, with questions one to three showing positive emotion scores and questions four to six showing negative emotion scores, both of which were distributed between 3 and 15. Based on the relevant definition of Subjective Wellbeing, the composite score of Subjective Wellbeing for each sample was calculated by subtracting the negative emotion score from the positive emotion score, and the range of the composite score was −12 to 12. For the convenience of calculation, a constant of 12 was added, and the range of scores was 0 to 24. The higher the score, the happier the self-perception, and the Cronbach α coefficient of the scale in this study was 0.71.

#### 2.2.4 Education level

According to the CLHLS questionnaire, “How many years of schooling have you attended in total?” is measured as years of formal education received. This was the moderating variable for the present study.

### 2.3 Data analysis

SPSS 21.0 was used for the statistical analysis of the data, and Mplus 8.0 was used for the statistical processing of the mediating and moderating effects analysis.

## 3 Results

### 3.1 Sample characteristics

Table 1 presents the characteristics of the entire study population. This study included 10,626 older adults (mean age: 83.39 ± 11.35 years). Among participants, there were 4,821 (45.4%) males and 5,805 (54.6%) females. There were 7,378 (69.4%) people with household registration in rural areas. There were 2,680 people (25.2%) currently living in the city, 3,514 people (33.1%) living in the town, and 4,432 people (41.7%) living in the countryside. Four thousand three hundred and one (40.5%) were aged 60–79 years, 5,898 (55.5%) were married, 8,517 (80.2%) lived with family members, 4,751 (44.7%) had not received education.

**Table 1:** Baseline characteristics for the study participants.

**Variables**	**Overall (*N =* 10,626)**	**Male (*N =* 4,821)**	**Female (*N =* 5,805)**	***X^2^***	***p***
Household registration				236.53	< 0.001
Urban areas	3,248 (30.6%)	1,110 (23.0%)	2,138 (36.8%)		
rural areas	7,378 (69.4%)	3,711 (77.0%)	3,667 (63.2%)		
Place of residence				331.22	< 0.001
City	2,680 (25.2%)	811 (16.80%)	1,869 (32.20%)		
Town	3,514 (33.1%)	1,797 (37.30%)	1,717 (29.60%)		
Rural	4,432 (41.7%)	2,213 (45.90%)	2,219 (38.20%)		
Age				1.179	0.143
60–79	4,301 (40.5%)	1,924 (39.9%)	2,377 (40.9%)		
More than 80	6,325 (59.5%)	2,987 (60.1%)	3,428 (59.1%)		
Marital status				3.41	0.03
Married	5,898 (55.5%)	2,723 (56.50%)	3,175 (54.70%)		
Unmarried, divorced, or widowed	4,728 (44.5%)	2,098 (43.50%)	2,630 (45.30%)		
Education year				1,305.98	< 0.001
0	4,751 (44.70%)	1,889 (39.20%)	2,862 (49.30%)		
1–6	3,620 (34.10%)	2,432 (50.40%)	1,188 (20.50%)		
7–12	1,823 (17.20%)	477 (9.90%)	1,346 (23.20%)		
13+	432 (4.10%)	23 (0.50%)	409 (7.00%)		
Co-residence				10.70	0.005
With household member(s)	8,517 (80.20%)	3,917 (81.20%)	4,600 (79.20%)		
Alone	1,732 (16.30%)	760 (15.80%)	972 (16.70%)		
In an institution	377 (3.50%)	144 (3.00%)	233 (4.00%)		

### 3.2 Common method deviation test

Because the data sources in this study were all derived from self-reported by participants, in order to avoid common methodology bias, Harman's single-factor test was used to perform unrotated exploratory factor analysis for all variables in this study (30). It was found that there were five common factors with characteristic roots >1, and the variance explained by the first common factor was 25.82%, which was much less than the critical value of 40%, indicating that there was no significant common method bias in this study.

### 3.3 Analysis of the correlation between variables

The older adult Subjective Wellbeing was significantly positively correlated with social participation and significantly negatively correlated with anxiety, as shown in Table 2.

**Table 2:** Correlation analysis between variables.

**Variables**	**1**	**2**	**3**	**4**	**5**	**6**	**7**	**8**
1. Gender	1							
2. Household type	−0.149^**^	1						
3. Place of residence	−0.143^**^	0.666^**^	1					
4. Age	0.018	0.048^**^	−0.009	1				
5. Subjective Wellbeing	0.172^**^	−0.156^**^	−0.123^**^	−0.163^**^	1			
6. Social participation	0.142^**^	−0.208^**^	−0.183^**^	−0.186^**^	0.509^**^	1		
7. Anxiety	−0.312^***^	0.116^**^	0.058^**^	0.048^**^	−0.571^**^	−0.350^**^	1	
8. Education	0.157^**^	−0.369^**^	−0.361^***^	−0.320^**^	0.296^**^	0.392^***^	−0.129^**^	1
M	1.55	1.69	2.16	83.39	16.34	17.36	1.55	3.66
SD	0.498	0.461	0.801	11.357	5.074	7.707	2.874	4.369

^***^p < 0.001, ^**^p < 0.01, ^*^p < 0.05.

### 3.4 The mediating role of anxiety

**Figure 1:**
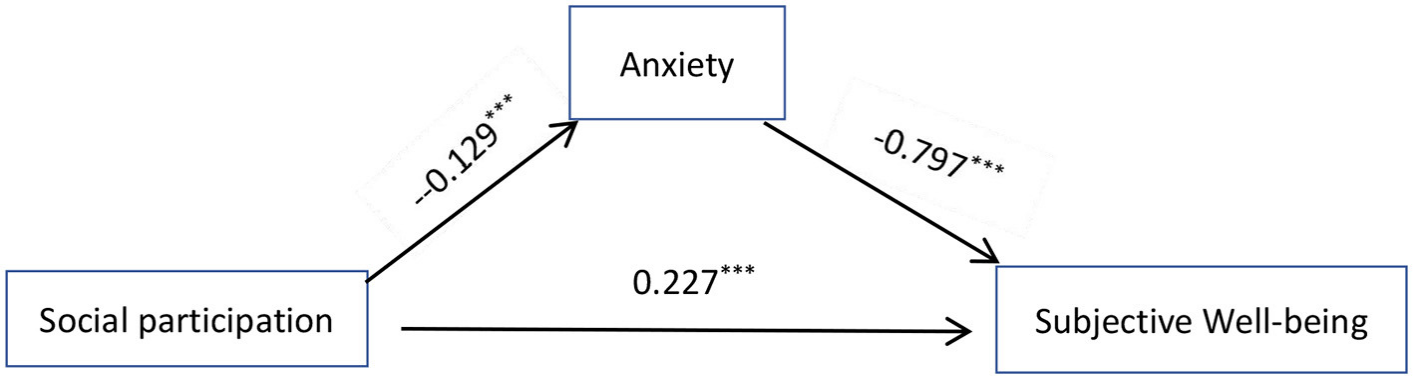
The mediating role of anxiety.

Structural equation modeling was constructed with Mplus 8.0 to examine the mediating role of anxiety between social participation and the older adult Subjective Wellbeing (see Figure 1). Gender, age, household types, and residence were used as control variables. The fit indices of the model were: χ^2^ = 103.807, df = 4, CFI = 0.969, TLI = 0.916, SRMR = 0.03, and RMSEA = 0.05, indicating that the model fits well. Bias-corrected non-parametric percentile Bootstrap tests were used to test the significance of mediating effects with 1,000 replicate samples. The results indicated that the 95% confidence interval for the mediating effect of anxiety between social participation and the older adult Subjective Wellbeing was (0.096, 0.109), excluding 0, indicating a significant mediating effect. The total effect of social participation on the older adult Subjective Wellbeing was 0.330, and the direct effect was 0.227, accounting for 62.73% of the total effect. The mediating effect of anxiety between social participation and Subjective Wellbeing was 0.103, accounting for 31.23% of the total effect, as detailed in Table 3.

**Table 3:** The mediation effect of anxiety.

**Effect**	**Paths**	**Effect value**	**Effect amount**	**95% confidence interval**
Direct effect	Social participation → Subjective Wellbeing	0.227	62.73%	
Intermediary effect	Social participation → Anxiety → Subjective Wellbeing	0.103	31.23%	(0.096, 0.109)
Total effect		0.330		

### 3.5 The moderating effects of education

**Figure 2:**
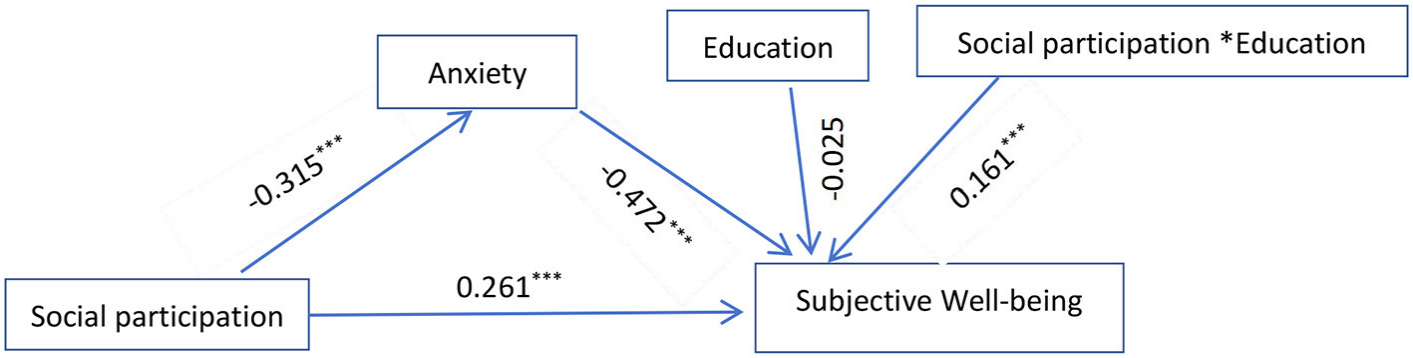
Moderating role of education.

The mediating effect model with adjustment was constructed by including gender, age, household types, and residence as control variables and education as moderating variables (see Figure 2). The fit indices of the model were: χ^2^ = 24.396, df = 2, CFI = 0.993, TLI = 0.976, SRMR = 0.017, RMSEA = 0.032, indicating that the model fits well. The results showed that the interaction term of education and social participation significantly predicted Subjective Wellbeing in the older adult (β = 0.161, *p* < 0.001). This indicated a significant moderating effect of education on the pathway of social participation and Subjective Wellbeing (see Table 4).

**Table 4:** Moderated mediation model testing.

**Predictive variable**	**M1 (Anxiety)**			**M2 (Subjective Wellbeing)**		
	β	***SE***	***t***	β	***SE***	***t***
Gender	−0.269	0.007	−36.841^***^	−0.034	0.008	−4.382^***^
Age	−0.01	0.009	−1.097	−0.060	0.008	−7.547^***^
Household types	0.072	0.012	6.134^***^	0.013	0.012	1.082
Residence	0.095	0.013	7.499^***^	−0.009	0.018	−0.498
Social participation	−0.315	0.008	−38.389^***^	0.261	0.015	17.608^***^
Anxiety				−0.472	0.015	−56.837^***^
Social participation ^*^Education				0.161	0.024	6.821^***^
R	0.443			0.430		
R^2^	0.196			0.185		
F	518.132			1,166.99		

^***^p < 0.001, ^**^p < 0.01, ^*^p < 0.05.

**Figure 3:**
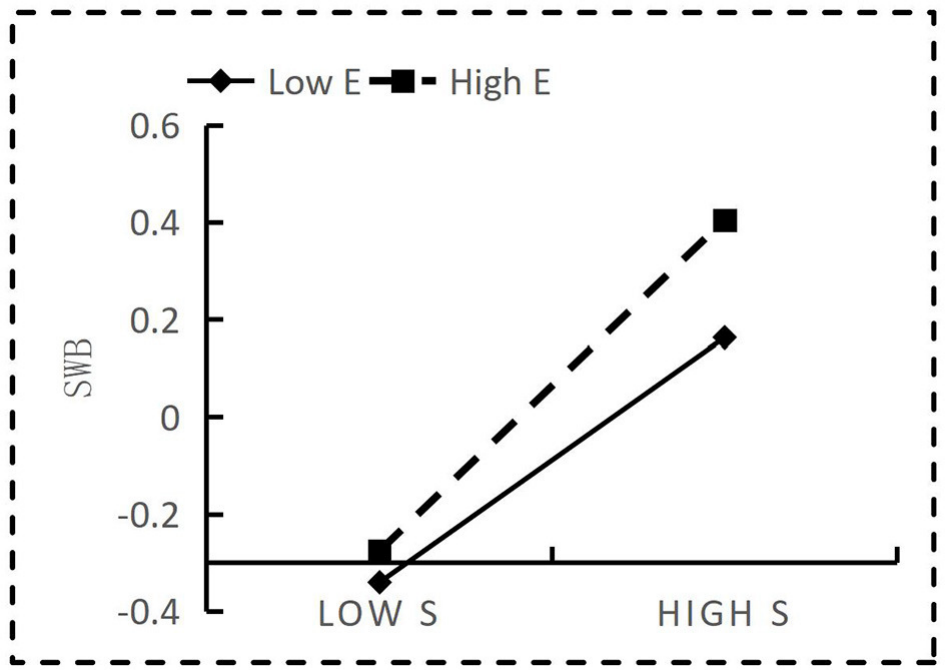
Moderating effect of education on social participation and Subjective Wellbeing (SWB, Subjective Wellbeing; E, education; S, social participation).

Further simple slope analysis demonstrated that social participation was a stronger predictor of Subjective Wellbeing for highly educated individuals, as shown in Figure 3 (b_simple_ = 0.175 *p* < 0.001) with a 95% confidence interval of [0.161, 0.175]. For low-educated individuals, social participation had a greater effect on the prediction of Subjective Wellbeing (b_simple_ = 0.162, *p* < 0.001) with a 95% confidence interval of [0.144, 0.178]. This result means that education positively moderates the relationship between social participation and Subjective Wellbeing by playing an amplifying role, indicating that when education is high, social participation enhancing impact on Subjective Wellbeing can increase.

## 4 Discussion

Using a large-scale sample, this study investigated the mechanisms of social participation on the Subjective Wellbeing of the older adult in China, the mediating role of anxiety, and the moderating role of education from the perspective of healthy aging. This research provides a foundation for promoting healthy living among the older adult.

In this study, it was found that social participation significantly predicts the Subjective Wellbeing of the older adult, which is consistent with previous results (13, 14). Active participation in various social activities can enhance the Subjective Wellbeing of the older adult. Social identity theory suggests that individuals can obtain an identity of being a member of a team through social participation, and this identity not only affects individual mental and health behaviors but also facilitates individuals to obtain social support in the team, thus enhancing the older adult's Subjective Wellbeing (31). The older adult's active participation in social activities such as playing cards, exercising, or engaging in leisure activities enhances their mental agility, maintains their physical fitness, and fosters life satisfaction through interaction with others (32). Ponce's study found that social participation promotes social integration and enhances life satisfaction among the older adult (33). Additionally, a study by Liao et al. on the older adult in China found that both high and low levels of social participation enhanced the older adult's Wellbeing. Social participation promoted the older adult's social interactions, helped made more friends, and increased their sense of social integration (34). This study further proved that the active participation of the older adult in social activities is a key factor in improving their Wellbeing.

This study revealed that anxiety was partially mediated between social participation and Subjective Wellbeing in the older adult in China, with a mediated effect value of 0.043, accounting for 13.48% of the total effect. High social participation can reduce anxiety in the older adult. The older adult without anxiety may face their old age in an optimistic way, and their Subjective Wellbeing will be higher. In the process of social participation, the older adult maintains interactions with friends and relatives, maintain harmonious interpersonal relationships, actively participate in social work or group activities, and realize their own value through social interaction and interpersonal communication. These activities can effectively reduce anxiety in the older adult (35). Social participation can also improve the older adult's social adaptation ability. Obtaining more social support from different channels, rationally understanding and accepting new social roles, and reducing anxiety due to withdrawal from the social stage or physical aging have positive effects on the physical and mental health of the older adult (36, 37). This study found that social participation is a protective factor against anxiety in the older adult. Subjective Wellbeing has two dimensions: emotional Wellbeing and life satisfaction. Emotional Wellbeing is a profound and stable long-lasting emotional experience closely related to the level of individual mental health (38). Social participation provides the older adult with stable, strong social interactions and emotional refuge, which is an important foundation for maintaining positive emotions and psychological Wellbeing late in life. On the basis of psychological health, the older adult is more likely to reap the benefits of happiness.

This study found that education moderated the pathway between social participation and the older adult Subjective Wellbeing. Specifically, the educated older adult social participation was a strong predictor of Subjective Wellbeing than the uneducated older adult. First, education can improve individuals' knowledge and cognitive ability. In old age, they are more willing to communicate with others, build a rich social support network, and participate in social activities, thus accumulating their social and spiritual resources. In turn, these spiritual resources can protect individuals' mental health and improve their Subjective Wellbeing (24). Second, highly educated individuals tend to be more receptive to healthy lifestyles, such as being physically active and engaging in leisure activities (39). They are able to construct high Subjective Wellbeing through these activities. Third, the educated older adult are likely to have higher social status and better social resources before retirement. This typically results in better social support when they need to talk or require assistance, higher self-efficacy, hope for life, and a sense of joy in life. This study examines the effects of social participation on the older adult Subjective Wellbeing, the mediating role of anxiety, and the moderating role of education from a healthy aging perspective.

This study found that anxiety partially mediates the relationship between social participation and the older adult Subjective Wellbeing and that education moderates the relationship between social participation and the older adult Subjective Wellbeing. After the older adult retires from the formal labor market, their emotional state will definitely be affected. Here are some implications for policy and practice. We can rely on the community to create more social participation opportunities for the older adult and organize various cultural and recreational activities and volunteer services so that the older adult can find social activities that meet their personal interests in their familiar living areas, enhance the diversity of their life in their old age, and thus improve their sense of Wellbeing and satisfaction. On the other hand, the community can also increase financial investment in education. For example, they can develop skill courses such as the use of electronic devices, knowledge courses such as the introduction of social hotspots and mental health knowledge, and interest courses such as sports and art instruction. By improving the educational level of the older adult, this can promote their social participation, enhance their sense of Wellbeing, and help maintain a healthy and happy life in their older adult years.

There are limitations in this study as follows: first, because this study is a cross-sectional study, there is no way to explore the causal relationship, and a longitudinal study can be added later to discuss the causal relationship between social participation and the older adult Subjective Wellbeing. Second, the study results were based on the self-reports from the participants, and recall bias or reporting bias for some questions could not be controlled.

## Data availability statement

The raw data supporting the conclusions of this article will be made available by the authors, without undue reservation.

## Author contributions

ZQ: Writing – original draft. CW: Writing – original draft, Formal analysis, Data curation. TG: Writing – review & editing.

## References

[1] Statistical Bulletin on National Economic and Social Development of the People's Republic of China in 2017 EB/OL. National Bureau of Statistics. (2018). Available online at: https://www.sohu.com/a/224489636_119663

[2] Strawbridge WJ Wallhagen MI Cohen RD. Successful aging and wellbeing. Gerontologist. (2002) 42:727–733. 10.1093/geront/42.6.727 12451153

[3] Um J Zaidi A Parry J Xiong Q. Capturing gendered aspects of active aging in china: insights drawn from the active aging index in comparison with EU countries. Asian Soc Work Policy Rev. (2021) 15:47–59. 10.1111/aswp.12218

[4] Greaves A. Leisure in later life (3rd edition). Aust Occup Ther J. (2006) 53:527. 10.1111/j.1440-1630.2006.00527.x

[5] Young Y Frick KD Phelan EA. Can successful aging and chronic illness coexist in the same individual? A multidimensional concept of successful aging. J Am Med Direct Assoc. (2009) 10:87–92. 10.1016/j.jamda.2008.11.003 19187875

[6] Diener E Suh EM Lucas RE Smith HL. Subjective Wellbeing: three decades of progress. Psychol Bull. (1999) 125:276. 10.1037//0033-2909.125.2.276

[7] Ryff CD. Happiness is everything, or is it? Explorations on the meaning of psychological wellbeing. J Person Soc Psychol. (1989) 57:1069–81. 10.1037//0022-3514.57.6.1069

[8] Huisingh-Scheetz M Wroblewski K Kocherginsky M Huang E Dale W Waite L. The relationship between physical activity and frailty among US older adults based on hourly accelerometry data. J Gerontol. (2018) 73:622–629. 10.1093/gerona/glx208 29106478 PMC5905616

[9] Carandang RR Shibanuma A Asis E Chavez DC Tuliao MT Jimba M. “Are Filipinos Aging Well?”: determinants of subjective well-being among senior citizens of the community-based ENGAGE study. Int J Environ Res Public Health. (2020) 17:7636. 10.3390/ijerph17207636 33092078 PMC7588882

[10] Hashidate H Shimada H Fujisawa Y Yatsunami M. An overview of social participation in older adults: concepts and assessments. Phys Ther Res. (2021) 24:85–97. 10.1298/ptr.R0013 34532203 PMC8419478

[11] McClelland AK. Self-conception and life satisfaction: integrating aged subculture and activity theory. J Gerontol. (1982) 37:723–32. 10.1093/geronj/37.6.723 7130647

[12] Han H Hengyuan Z Yonggang T. Patterns of social participation and impacts on memory among the older people. Front Public Health. (2022) 10:963215. 10.3389/fpubh.2022.963215 36457313 PMC9706236

[13] Zhao X Liu H Fang B Zhang Q Ding H Li T. Continuous participation in social activities as a protective factor against depressive symptoms among older adults who started high-intensity spousal caregiving: findings from the China health and retirement longitudinal survey. Aging Ment Health. (2021) 25:1821–9. 10.1080/13607863.2020.1822283 32954798

[14] Fang B Huang J Zhao X Liu H Chen B Zhang Q. Concurrent and lagged associations of social participation and frailty among older adults. Health Soc Care Commun. (2022) 30:E4812–20. 10.1111/hsc.13888 35717629

[15] Gang M Ko H Lee J. Aging anxiety and subjective wellbeing of persons with mental disorder. Korea Contents Assoc. (2019) 19:329–38. 10.5392/JKCA.2019.19.01.329

[16] Schwarz R Gunzelmann T Hinz A Brähler E. Anxiety and depression in the general population over 60 years old. Deutsche Med Wochenschrift. (2001) 126:611–5. 10.1055/s-2001-14420 11413747

[17] Guner TA Erdogan Z Demir I. The effect of loneliness on death anxiety in the older adult during the COVID-19 pandemic. OMEGA-J Death Dying. (2023) 87:262–82. 10.1177/00302228211010587 33878967 PMC8060692

[18] Ida S Murata K. Social participation benefit in older adult patients with diabetes: a scoping review. Gerontol Geriatr Med. (2022) 8:1–8. 10.1177/23337214221093887 35464637 PMC9021472

[19] Yue Z Liang H Gao X Qin X Li H Xiang N. The association between falls and anxiety among older adult Chinese individuals: the mediating roles of functional ability and social participation. J Affect Disor. (2022) 301:300–6. 10.1016/j.jad.2022.01.070 35051441

[20] Evans IE Llewellyn DJ Matthews FE Woods RT Brayne C Clare L. Social isolation, cognitive reserve, and cognition in older people with depression and anxiety. Aging Ment Health. (2019) 23:1691–700. 10.1080/13607863.2018.1506742 30518250

[21] Chen Y Lv C Li X Zhang J Chen K Liu Z. The positive impacts of early-life education on cognition, leisure activity, and brain structure in healthy aging. Aging (Albany NY). (2019) 11:4923. 10.18632/aging.102088 31315089 PMC6682517

[22] Mazzonna F. The long-lasting effects of family background: a European cross-country comparison. Econ Educ Rev. (2014) 40:25–42. 10.1016/j.econedurev.2013.11.010

[23] Xiaolin L Chong Z. A study on the influence of social participation on the health of the older adult: from the perspective of urban-rural differences. J Xihua Univ. (2023) 42:57–71. 10.12189/j.issn.1672-8505.2023.02.008

[24] Mazzonna F. The long lasting effects of education on old age health: evidence of gender differences. Soc Sci Med. (2014) 101:129–38. 10.1016/j.socscimed.2013.10.042 24560233

[25] Pothisiri W Prasitsiriphon O Saikia N Aekplakorn W. Education and grip strength among older Thai adults: a mediation analysis on health-related behaviours. SSM-Popul Health. (2021) 15:100894. 10.1016/j.ssmph.2021.100894 34458550 PMC8379495

[26] Zhongjun D. Health education and the improvement of old people's happiness index. Health Educ Health Promot. (2012) 7:232–4. 10.16117/j.cnki.31-1974/r.2012.03.02432698473

[27] Shan Q Li S. Diagnostic test of screening generalized anxiety disorders in general hospital psychological department with GAT-7. Chin Mental Health J. (2015) 29:939–44. 10.3969/j.issn.1000-6729.2015.12.010

[28] Bradburn NM. The structure of psychological wellbeing. Soc Serv Rev. (1969) 76:44. 10.1037/t10756-000

[29] Watson D Clark LA Tellegen A. Development and validation of brief measures of positive and negative affect: the panas scales. J Pers Soc Psychol. (1988) 54:1063–70. 10.1037//0022-3514.54.6.1063 3397865

[30] Dandan T Zhonglin W. Statistical approaches for testing common method bias: problems and suggestion. J Psychol Sci. (2020) 43:215–23. 10.16719/j.cnki.1671-6981.20200130

[31] Jetten J Haslam C Haslam SA Dingle G Jones JM. How groups affect our health and well-being: the path from theory to policy. Soc Issues Policy Rev. (2014) 8:103–30. 10.1111/sipr.12003

[32] Uchino BN. Social support and health: a review of physiological processes potentially underlying links to disease outcomes. J Behav Med. (2006) 29:377–87. 10.1007/s10865-006-9056-5 16758315

[33] Ponce MS Rosas RP Lorca MB. Social capital, social participation and life satisfaction among Chilean older adults. Rev Saude Publica. (2014) 48:739–49. 10.1590/S0034-8910.2014048004759 25372164 PMC4211572

[34] Liao Z Zhou H He Z. The mediating role of psychological resilience between social participation and life satisfaction among older adults in China. BMC Geriatr. (2022) 22:948. 10.1186/s12877-022-03635-x 36482364 PMC9733394

[35] Choi E Han KM Chang J Lee YJ Choi KW Han C. Social participation and depressive symptoms in community-dwelling older adults: emotional social support as a mediator. J Psychiatr Res. (2021) 137:589–96. 10.1016/j.jpsychires.2020.10.043 33168196

[36] Wang X. Subjective Wellbeing associated with size of social network and social support of older adult. J Health Psychol. (2016) 21:1037–42. 10.1177/1359105314544136 25104778

[37] Kienko T Rudakova R. The Subjective Wellbeing of older adult residents in Russian nursing homes. J Soc Policy Stud. (2020) 18:255–68. 10.17323/727-0634-2020-18-2-255-268

[38] Choudhary A Pathak A Manickam P Purohit M Rajasekhar TD Dhoble P. Effect of yoga versus light exercise to improve well-being and promote healthy aging among older adults in central India: a study protocol for a randomized controlled trial. Geriatrics. (2019) 4:64. 10.3390/geriatrics4040064 31744171 PMC6960920

[39] Cutler DM Lleras-Muney A. Understanding differences in health behaviors by education. J Health Econ. (2010) 29:1–28. 10.1016/j.jhealeco.2009.10.003 19963292 PMC2824018

